# Identifying and Quantifying Neurological Disability via Smartphone

**DOI:** 10.3389/fneur.2018.00740

**Published:** 2018-09-04

**Authors:** Alexandra K. Boukhvalova, Emily Kowalczyk, Thomas Harris, Peter Kosa, Alison Wichman, Mary A. Sandford, Atif Memon, Bibiana Bielekova

**Affiliations:** ^1^Laboratory of Clinical Immunology and Microbiology, Neuroimmunological Diseases Section, National Institute of Allergy and Infectious Diseases (NIAID), National Institutes of Health (NIH), Bethesda, MD, United States; ^2^Department of Computer Science, University of Maryland, College Park, MD, United States

**Keywords:** smartphone app, diagnostics, medical technology, precision medicine, multiple sclerosis, neurology, outcomes, neurological examination

## Abstract

Embedded sensors of the smartphones offer opportunities for granular, patient-autonomous measurements of neurological dysfunctions for disease identification, management, and for drug development. We hypothesized that aggregating data from two simple smartphone tests of fine finger movements with differing contribution of specific neurological domains (i.e., strength & cerebellar functions, vision, and reaction time) will allow establishment of secondary outcomes that reflect domain-specific deficit. This hypothesis was tested by assessing correlations of smartphone-derived outcomes with relevant parts of neurological examination in multiple sclerosis (MS) patients. We developed MS test suite on Android platform, consisting of several simple functional tests. This paper compares cross-sectional and longitudinal performance of Finger tapping and Balloon popping tests by 76 MS patients and 19 healthy volunteers (HV). The primary outcomes of smartphone tests, the average number of taps (per two 10-s intervals) and the average number of pops (per two 26-s intervals) differentiated MS from HV with similar power to traditional, investigator-administered test of fine finger movements, 9-hole peg test (9HPT). Additionally, the secondary outcomes identified patients with predominant cerebellar dysfunction, motor fatigue and poor eye-hand coordination and/or reaction time, as evidenced by significant correlations between these derived outcomes and relevant parts of neurological examination. The intra-individual variance in longitudinal sampling was low. In the time necessary for performing 9HPT, smartphone tests provide much richer and reliable measurements of several distinct neurological functions. These data suggest that combing more creatively-construed smartphone apps may one day recreate the entire neurological examination.

## Introduction

Neurological examination measures diverse functions of the central (CNS) and peripheral nervous systems to diagnose neurological diseases and guide treatment decisions. Thorough neurological examination takes between 30 and 60 min to complete and years of training to master. This poses problem both for developing countries, which often lack neurologists, and for developed countries where cost-hikes and administrative requirements severely limit the time clinicians spend examining patients.

Additionally, clinical scales derived from traditional neurological examination are rather insensitive and prone to biases, which limits their utility in drug development. Therefore, non-clinician administered measurements of physical disability such as timed 25-foot walk (25FW) and 9-hole peg test (9HPT) or measurements of cognitive functions exemplified by paced auditory serial addition test (PASAT) and symbol digit modalities test (SDMT), are frequently used in clinical trials of neurological diseases such as multiple sclerosis (MS) (1, 2). Especially combining these “functional scales” with clinician-based disability scales such as Expanded Disability Status Scale (EDSS)(3) into EDSS-plus (4) or Combinatorial weight-adjusted disability scale (CombiWISE) (5) enhances sensitivity of clinical trial outcomes. However, these sensitive combinatorial scales are rarely, if ever acquired in clinical practice due to time and expense constrains.

Measuring neurological functions by patients via smartphones (6–8) may pose a solution for all aforementioned problems, while additionally empowering patients for greater participation in their neurological care. We have previously found comparable sensitivity and specificity of simple, smartphone-amenable measurements of finger and foot taps to 9HPT and 25FW, respectively (9). In this study, we explored iterative development/optimization of smartphone-based measurements of neurological functions by: 1. Exploring clinical utility of new features that can be extracted from finger tapping; 2. Development of “balloon popping” smartphone test that builds on finger tapping by expanding neurological functions necessary for task completion to eye movements and cognitive skills, and 3. By decoding app-collected raw data into secondary (derived) features that may better reflect deficits in specific neurological functions.

## Materials and methods

### Developing the smartphone apps

Tapping and Balloon popping tests were written using Java in the Android Studio integrated development environment. Both tests went through iterative development and optimization following beta testing with developers and then clinical trial testing with patients and healthy volunteers. Each of the individual tests are standalone applications and can be downloaded individually to the phone using an Android Package (APK) emailed to phones or directly installed through USB connection with Android Studio. Installation and initial testing of applications were completed on a variety of personal Android phones, with no particular specifications. Testing in the clinic with patients and longitudinal testing was completed on Google Pixel XL 2017 phones. Android 8.1 Oreo operating system was used for the most recent version of the application, with the intention of keeping the operating system the app runs on up to date with the most recent version released by Android.

For the purposes of this study, we created a front-end application that can flexibly incorporate a variety of test apps. The front-end prompts for user profiles where a testing ID, birth month and year, gender, and dominant hand may be entered so data collected is associated with the user profile. Through a cloud-based spreadsheet, “prescriptions” of test app configurations are set for each user such that they may have a unique combination of tests tailored to their disability level.

**Figure 1:**
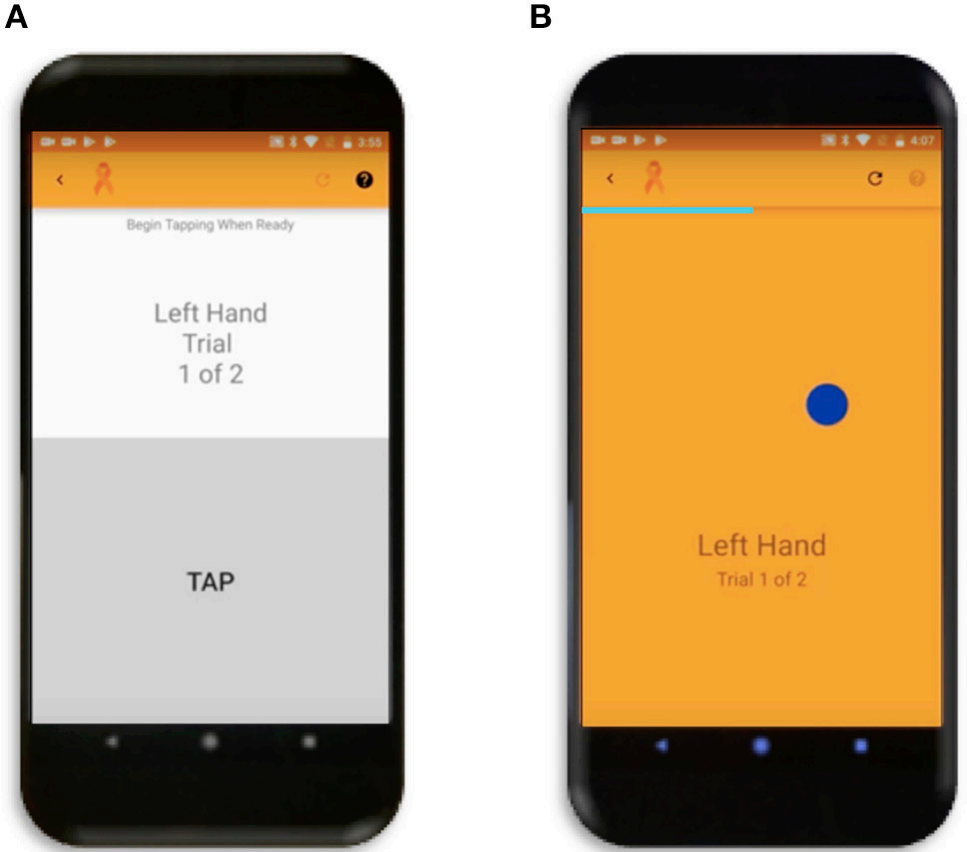
Smartphone Apps. (**A)** Tapping Test where user can tap repeatedly anywhere in the gray rectangle over the bottom half of the screen. (**B)** Popping Test where the dark blue circle will disappear and simultaneously reappear randomly across the screen as soon as the user touches it.

The tapping test goal was similar to previously validated non-smartphone administered tapping tests (9), where users had to tap as quickly as possible over a 10 s duration and the final score is the average of two attempts. The test uses touch recognition over a rectangular area covering the bottom half of a vertically oriented phone screen (Figure 1A). Users can tap anywhere in a marked off gray area. The total number of taps for each of two trials and the calculated average is displayed immediately afterwards on the screen. In addition to total taps over the duration of the test, the app also records the duration, Android system time, and pressure for each tap as background data. Pressure for app recording is interpreted from the size of the touch area on each tap, where larger tap area corresponds to a higher pressure reading. Because the pressure function was added later and therefore the data are missing for the majority of current cohort, this function is not investigated in current study.

The balloon popping test was conceptually envisioned as an extension of tapping test that expands neurological functions necessary for test completion from pure motoric, to motoric, visual, and cognitive (attention and reaction time). The primary goal for this test is to touch as many randomly generated dark blue circles (balloons) moving across the screen in succession over the 26-s test duration as possible. During optimization of the app we tested 3 sizes of the target balloon and a 100-pixel balloon was selected as optimal based on preliminary results. The analyses of the other two circle sizes are provided as part of sensitivity analyses (Supplementary Figure 1), as conclusions from these tests support data presented in the main text of the paper. There is only one balloon to pop on the screen at a time (Figure 1B) and as soon as the user touches anywhere on the circle, another circle will appear in a random location. The random generation of balloon locations was created by random number functions in Java for both the x and y coordinates of the center of the circle, with the constraint of the entire balloon having to be visible on the screen. If the user taps on a background location, the current balloon stays in the same location and is only moved to a new random location after accurately tapping on the balloon. Following app completion, the total number of balloons popped and calculated average (from two trials) is displayed on the phone for the user. The x and y coordinates of all balloon and background hits, the system time, duration, and pressure (in the same manner as tap pressure) for each tap are also recorded as background data and stored in cloud-based data system.

Following the completion of a tapping or balloon popping test trial, an intermediate message displayed on the screen asks if the users would like to submit their results or retake the most recent trial (Supplementary Videos 1, 2). If the user selects the retake option the collected data for the trial is discarded locally on the phone and not sent to any cloud-based database. This was implemented to avoid noise associated with test interruptions or other unforeseen circumstances that affected test performance. Following selection of the submit option, the data is uploaded immediately to a cloud-based database if the smartphone is connected to WiFi. If the phone is not connected to WiFi, then the submitted test trial results are stored locally on the phone and uploaded to the database as soon as the phone is connected to WiFi.

The app development process is in continuation given user and clinician feedback in addition to integration of more tests into the front-end. User feedback, user's ability to perform Apps in a “practice mode”, and training videos for individual tests (Supplementary Videos 1, 2) are integrated into the front-end dashboard that manages different tests.

### Patient populations

Data was collected from healthy volunteers (HV) and multiple sclerosis patients (MS) participating in protocols: Comprehensive multimodal analysis of neuroimmunological diseases of the CNS (Clinical trials.gov identifier NCT00794352) and Targeting Residual Activity by Precision, biomarker-guided combination therapies of Multiple Sclerosis (TRAP-MS; NCT03109288). All protocols were approved by the NIH Combined Neuroscience Institutional Review Board and all participants provided informed consent. A total of 19 HV and 76 MS participants completed cross sectional data, entailing 2 trials of both tapping and balloon popping tests on their first test sitting (Table 1). Out of these, 15 HV, and 16 MS patients provided also longitudinal data with median of 7 independent (i.e., on separate days) trials for HV and 9 weekly trials for MS patients (Table 2). The majority of HVs participated in the Smartphone app-only sub-study of the NCT00794352 protocol, which keeps their identity anonymous. Only self-reported age and gender are recorded in the smartphone app.

Table 1Demographics for cross sectional participants.

**Table:** 

**Cohort**	**N**	**Female (%)**	**Average disease duration**	**Average age**	**Average NeurEx**	**Average EDSS**
**(A)**						
Total MS	76	56.58	16.71	56.54	138.40	4.91
PP-MS	35	54.29	15.63	60.12	168.22	5.62
SP-MS	18	61.11	25.27	59.37	175.26	5.92
RR-MS	23	56.52	11.54	48.86	64.17	3.09

**Table:** 

**Cohort**	**N**	**Female (%)**	**Average disease duration**	Average NeurEx^*^	Average EDSS^*^
**(B)**					
Total MS	76	56.58	16.71	138.40	4.91
18–29	0	–	–	–	–
30–39	4	75.00	9.16	47.85	2.62
40–49	13	61.54	10.58	88.82	3.85
50–59	28	53.57	15.10	138.57	4.96
>60	31	54.84	21.70	170.71	5.61

**Table:** 

**Cohort**	**N**	**Female (%)**	Average NeurEx^*^	Average EDSS^*^
**(C)**				
Total HV	19	50.00	20.12	1.50
18–29	6	50.00	–	–
30–39	3	33.33	10.00	1.00
40–49	3	66.67	11.45	1.50
50–59	1	100.00	15.07	1.25
>60	6	50.00	43.22	2.00

***(A)** Multiple Sclerosis (MS) test participants by disease type cohort including primary progressive (PP-MS), secondary progression (SP-MS), and relapsing-remitting (RR-MS)*.

***(B)** MS split by age cohort. **(C)** Healthy Volunteer (HV) participants split by age cohort*.

* *10 of the HV participants were part of the natural history protocol for the clinical trials our MS patients participated in and had documented NeurEx and EDSS scores*.

Table 2Demographics for longitudinal participants.

**Table:** 

**Cohort**	***N***	**Female (%)**	**Average disease duration**	**Average age**	**Average NeurEx**	**Average EDSS**
**(A)**						
Total MS	16	62.50	21.48	60.97	164.86	5.31
PP-MS	10	70.00	18.56	62.40	169.95	5.35
SP-MS	5	60.00	30.37	59.59	175.88	5.60
RR-MS	1	0.00	6.23	53.61	58.96	3.50

**Table:** 

**Cohort**	***N***	**Female (%)**	**Average disease duration**	Average NeurEx^*^	Average EDSS^*^
**(B)**					
Total MS	16	62.50	21.48	164.86	5.31
18-29	–	–	–	–	–
30-39	–	–	–	–	–
40-49	–	–	–	–	–
50-59	6	50.00	22.65	144.72	5.17
>60	10	70.00	20.78	176.95	5.40

**Table:** 

**Cohort**	**N**	**Female (%)**	Average NeurEx^*^	Average EDSS^*^
(**C**)				
Total HV	15	46.67	18.82	1.50
18–29	6	50.00	–	–
30–39	0	–	–	–
40–49	2	50.00	12.90	1.50
50–59	1	100.00	15.07	1.25
>60	5	60.00	32.24	2.00

***(A)** Multiple Sclerosis (MS) test participants by disease type cohort including primary progressive (PP-MS), secondary progression (SP-MS), and relapsing-remitting (RR-MS)*.

*(**B)** MS participants split by age cohort. **(C)** Healthy Volunteer (HV) participants split by age cohort*.

* *4 of the HV participants were part of the natural history protocol for the clinical trials our MS patients participated in and had documented NeurEx and EDSS scores*.

### App testing

Both HV and MS participants completed all cross-sectional testing under the supervision of lab personnel who explained the overarching goal of the tapping and balloon popping tests. Participants could complete the tests on practice mode prior to data recording. Participants enrolled in longitudinal testing were instructed on how to complete testing on their own and instructed to complete tests around the same time of day on their own schedule (i.e., morning testing vs. afternoon testing).

### Neurological examination and neurex score

MS-trained clinicians examined all MS patients at the time of their first (cross-sectional) smartphone apps testing and documented EDSS (3) into Neuroimmunological Diseases Section (NDS) research database and NIH electronic medical records. Additionally, the neurological examination in its entirety was documented electronically (via iPad or desktop) into NeurEx app developed by NDS (10) and linked to NDS database. NeurEx app automatically computes four disability scales used in neuroimmunology, including EDSS, and Kurtzke Functional Systems Scores (FSS). NeurEx app also provides summary NeurEx score, which ranges from 0 to 1,349 with 0.25 minimal measurable step. This digitalization of neurological examination generates limb-specific and neurological function-specific sub scores, against which we correlated smartphone app results. 9HPT, SDMT, and PASAT results were collected at the time of neurological examination by support staff.

### Data processing and statistical analysis

Data was stored in cloud-based database and continuously updated with streamed results. Raw datasheets were manually pulled and then processed using Python. We analyzed two cohorts: Cross-sectional cohort was defined as the first date when the user completed two trials for both the tapping and balloon popping tests. We pre-processed data to eliminate outliers: for cross-sectional data composed of two trials we excluded the worst trial if the ratio between the two trials was between 0.5 and 2 (i.e., the worse trial was at least two times worse than the better trial). We defined the total number of taps and pops in a trial as simple app feature. For simple features, results from the cohorts were compared between dominant, non-dominant, and averaged hand data.

Difference between HV and MS patients' data was assessed by Wilcoxon Rank-Sum tests producing the test statistic (U) and two-sided *p*-value with the null hypothesis that the sets of measurements for each cohort are drawn from the same distribution. Pearson correlations were completed between clinical trial participants' simple feature scores, 9HPT scores, and relevant clinician rated clinical scale scores. Correlation coefficients (R) and the associated two-sided *p*-values were based on the null hypothesis that the correlation between the two sets of measurements was 0.

Longitudinal analysis data was plotted for each patient completing more than 2 unique test sittings. Intra-individual outlier tests that exceeded 1.5^*^ interquartile range (IQR) calculated for each individual longitudinal tester features were excluded. Tapping and balloon popping scores were directly compared over time for each patient by normalizing all scores to per-second scores. All scores for both tests were converted to a percentage of the HV median score for the respective test.

Due to collinearity in most of the clinical and functional outcomes we did not employ Bonferroni correction for multiple comparisons. Instead, we used a *p*-value of <0.01 as a cut-off for statistical significance, but provide either raw *p*-values or their range.

## Results

### Cross sectional data

#### Self-administered finger tapping and balloon popping have comparable power for detecting significant differences between MS patients and HV with traditional, investigator-administered 9HPT

**Figure 2:**
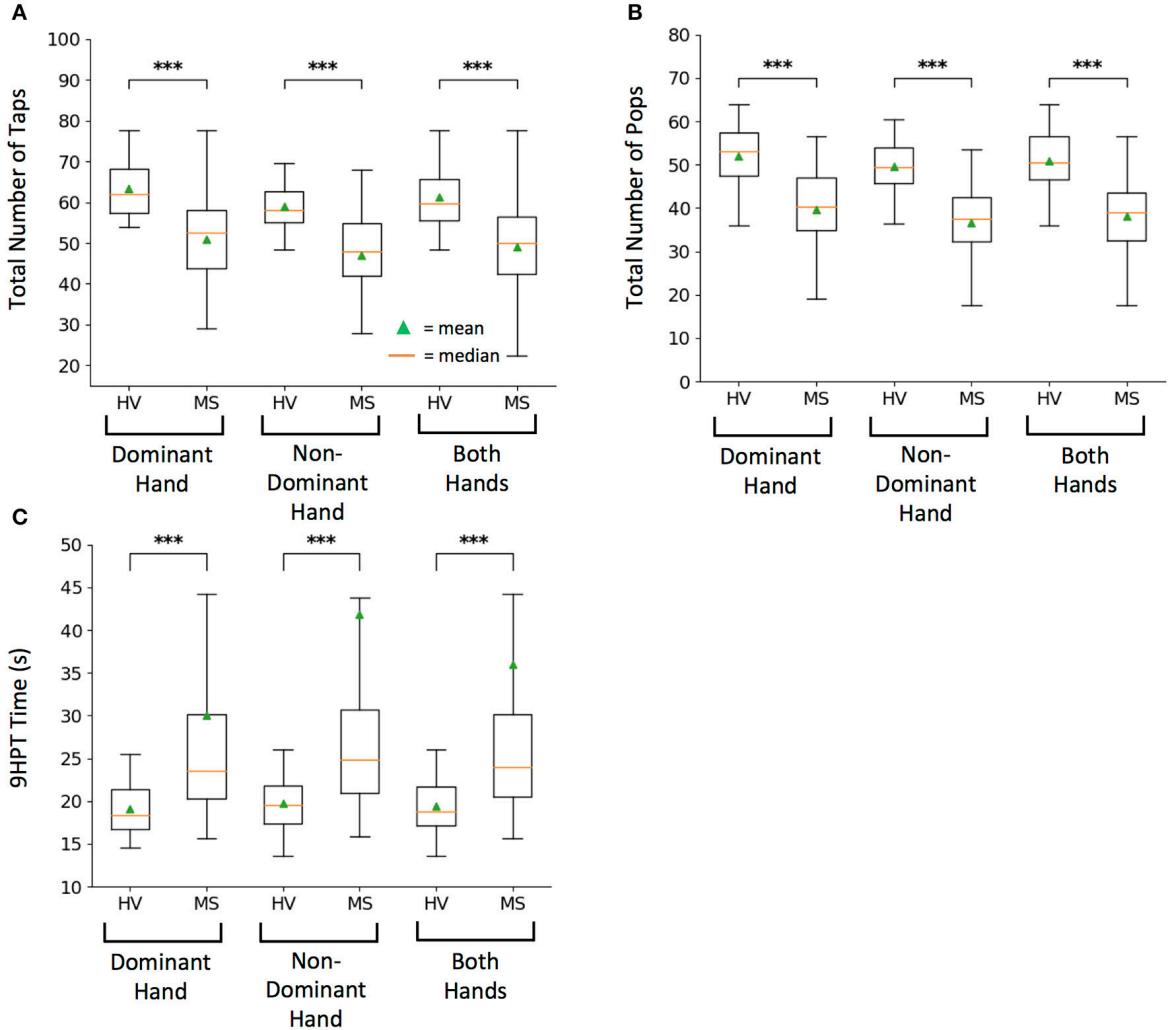
Healthy volunteer (HV) and Multiple Sclerosis (MS) cohort comparison for raw scores on tests. **(A)** Smartphone-developed tapping test measuring total number of taps in 10 s. **(B)** Smartphone-developed balloon popping test measuring total number of randomly generated balloons popped in 26 s. **(C)** Current clinical motor function exam, 9-hole peg test (9HPT) measuring time for completion. Lower 9HPT time is considered the better outcome. For each test, box plots represent group values based on diagnosis, where each subject is represented by an average of two independent attempts, with intra-individual outliers excluded as described in methods. On each box plot, green triangles indicate the mean and orange lines indicate the median of depicted cohort datasets. Box plot box boundaries represent the Q1 to Q3 range centered about the median. Box plot whisker lengths extend to Q1–1.5 *IQR and Q3 + 1.5 *IQR. ***p < 0.0001.

The total numbers of taps for both hands differentiated MS from HV most significantly (*U* = 6.11, *p* = 1.00e−09) as compared to dominant (*U* = 4.31, *p* = 1.62e−05) and non-dominant (*U* = 4.45, *p* = 8.64e−06) hand taps (Figure 2A).

Balloon popping test had even greater discriminatory power, with averaged data for both hands (*U* = 6.81, *p* = 1.00e−11) leading over dominant (*U* = 4.62, *p* = 3.85e−06) and non-dominant hand scores (*U* = 5.22, *p* = 1.80e−07) (Figure 2B).

Both primary features of the smartphone tests differentiated MS from HV with lower power than 9HPT. This was true for averaged data of both hands (*U* = −8.17, *p* = 3.09e−16), as well as for dominant (*U* = −5.66, *p* = 1.49e−08) and non-dominant hand scores (*U* = −5.93, *p* = 3.00e−09; Figure 2C). However, this difference in power included 4 MS patients who were no longer able to perform 9HPT and their failed attempts were coded according to guidelines (1) as 300 s, which represents outlier score in comparison to maximum true achievable score of 299 s. In contrast all patients could perform tapping and balloon popping tests, demonstrating higher “ceiling effect” threshold for smartphone app tests.

As previously seen for 9HPT (5), non-dominant hand scores had slightly higher power in differentiating MS from HVs than dominant hand scores also for smartphone app-based scores.

#### Smartphone outcomes outperform 9HPT in correlating with relevant subscores of digitalized neurological examination performed by MS-trained clinicians

To assess concordance between self-administered and investigator-administered functional tests, we correlated tapping, and balloon popping scores with 9HPT and with the appropriate-limb-specific sub scores of the neurological examinations performed by a MS-trained clinician.

**Figure 3:**
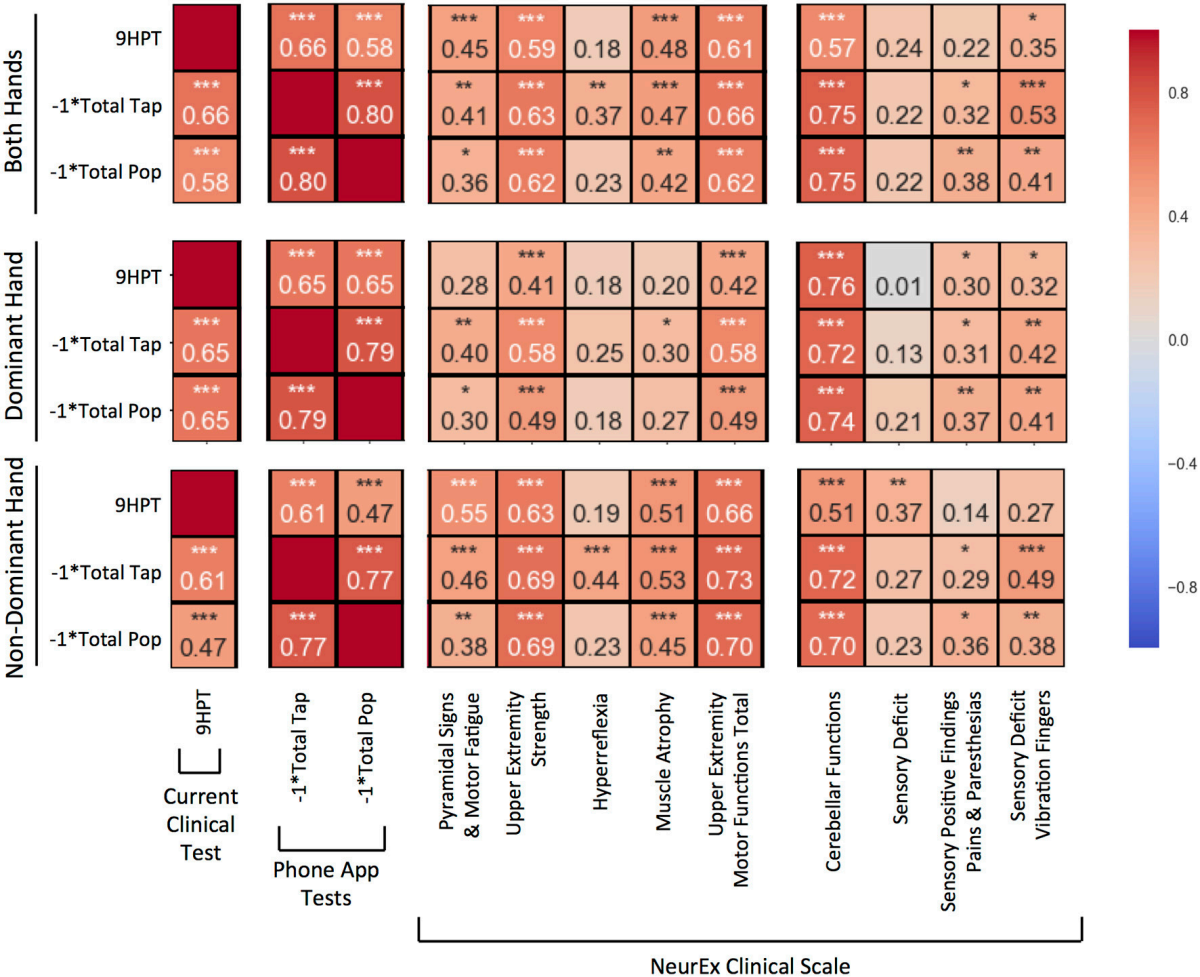
Pearson correlations compared between 9-hole peg test (9HPT), inverse scores of the Tap and Balloon Popping tests, and clinician-derived scores from NeurEx. The score for “Upper Extremity Motor Functions Total” is a combination of the previous 5 scores to the left in the same sectioned off block. Red to blue heat map represents value of the correlation coefficient ranging from 1 to −1, respectively. *p < 0.01, **p < 0.001, ***p < 0.0001.

For each hand, the correlations between total number of taps and pops were stronger than correlations between smartphone apps and 9HPT. Nevertheless, correlations of smartphone tests with 9HPT were still highly statistically significant (*p* < 0.0001) and of moderate (*R* = 0.4–0.5) to high (*R* > 0.5) strength (Figure 3).

The new, smartphone-based outcomes outperformed traditional 9HPT in correlation analyses with clinician-derived outcomes, with balloon popping test achieving the strongest correlations. This was true for virtually all comparisons (i.e., for dominant, non-dominant hand, and the average of both hands).

Specifically, for average of both hands (Figure 3, upper panel), the popping scores had the strongest and most significant (*p* < 0.0001) correlations with upper extremity strength (*R* = 0.62), cerebellar functions (*R* = 0.75), and the overall upper extremities motor exam (*R* = 0.62) that integrates muscle strength with hyperreflexia, pyramidal signs, and muscle atrophy. Somewhat lower correlations, but nevertheless highly significant, were observed with the quantitative measure of proprioception, vibration sense. Specifically, we measured decay of vibration by 128 Hz tuning fork in fingers of each hand and observed that the inverse of the decay correlated significantly (*p* < 0.001) with popping scores (*R* = 0.41). Tapping scores also had highly statistically significant correlations with analogous subscores of the neurological examination (*R* = 0.63, 0.75, 0.66, and 0.53). The weakest, but still highly significant correlations with aforementioned neurological functions were observed for 9HPT (*R* = 0.59, 0.57, 0.61, and 0.35).

Analogous results were observed for individual hands (Figure 3, middle and lower panels), with dominant hand exerting stronger correlations with cerebellar functions and non-dominant hand having slightly stronger correlations with motoric functions.

#### Digitalization of functional tests allows development of novel (secondary) outcomes that may better reflect specific neurological dysfunctions

The digitalization of the functional tests allows collection of additional features, not available for currently used mechanical version of the 9HPT, such as variance of time between the taps (or pops). We explored 3 features of potential clinical significance derived from these secondary digital data.

##### Cerebellar dysfunction

We hypothesize that the cerebellar dysfunction (ataxia and dysdiadochokinesis) will increase the variance of time between the taps and/or variance of “dwell” time, which is the time the finger is in the contact with the screen. Therefore, we explored these variance parameters as a measure of cerebellar dysfunction. The goal of this exploration was to derive a score that could at least partially dissociate motor weakness/spasticity (that affects more the speed of test performance) from cerebellar dysfunction (that may exert larger influence on the variance of test performance).

**Figure 4:**
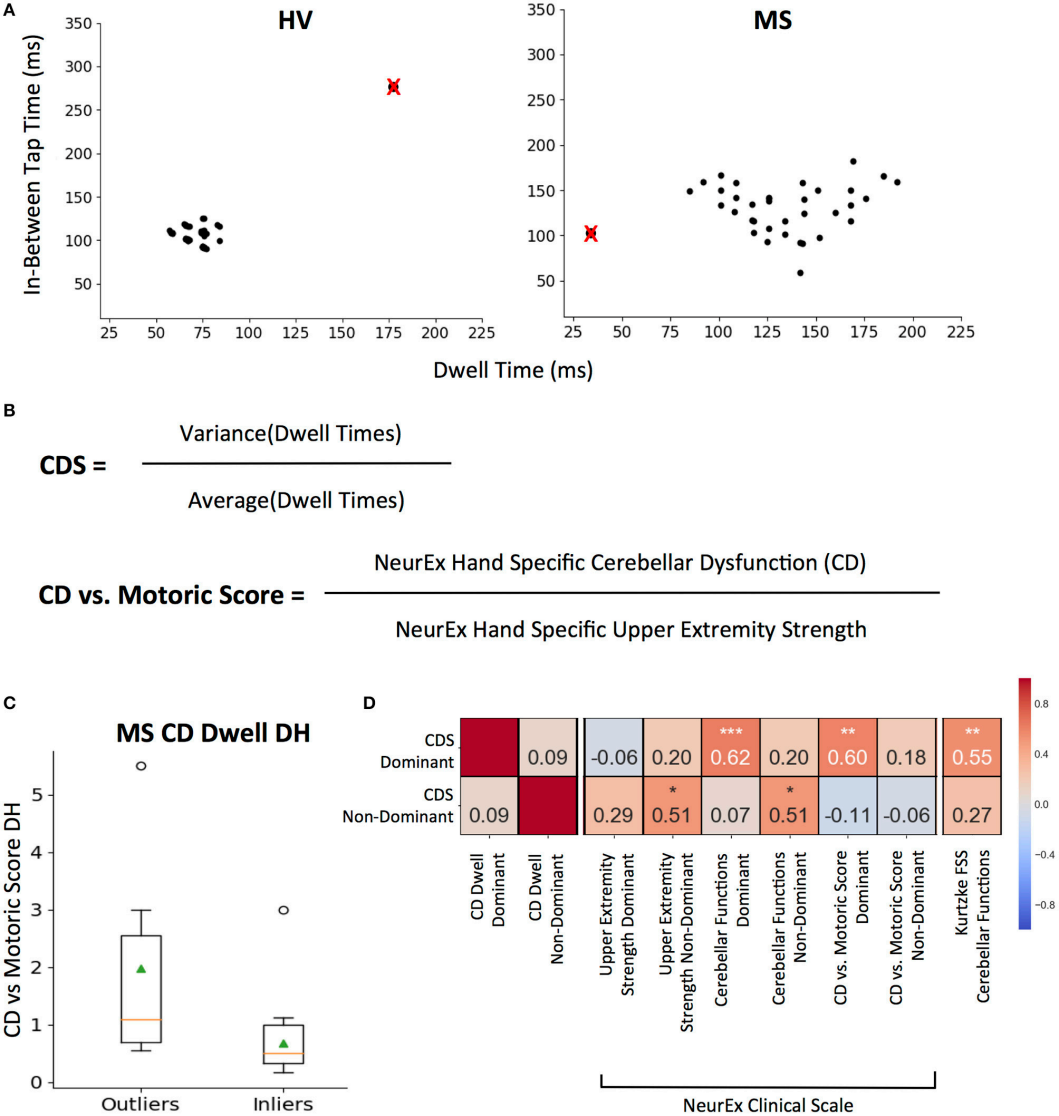
CDS: Cerebellar Dysfunction Score designed to better separate cerebellar from motoric disability. **(A)** Representative tapping patterns for Healthy Volunteer (HV) participant and Multiple Sclerosis (MS) participants. Individual plot is of a 10 s tap trial, with the corresponding dwell time and in-between tap time for each tap. The black points with a red “x” represents example outlier taps for dwell time and/or in-between tap time that is eliminated based on IQR thresholds as defined in the methods. **(B)** Generating cerebellar dysfunction score (CDS) based on dwell times to quantify tapping regularity. The score for each tapping trial is the coefficient of variation of inlier dwell times. Generating Cerebellar Dysfunction (CD) vs. Motoric dysfunction clinical score by comparing NeurEx scores for hand specific cerebellar dysfunction with upper extremity strength. **(C)** Distribution of Cerebellar vs. Motoric scores between MS groups that were outliers and inliers for the CDS. On each box plot, green triangles indicate the mean and orange lines indicate the median of depicted cohort datasets. Box plot box boundaries represent the Q1 to Q3 range centered about the median. Box plot whisker lengths extend to Q1–1.5 *IQR and Q3 + 1.5 *IQR. **(D)** Pearson correlations between CDS for dominant and non-dominant hands and corresponding clinical scores. Red to blue heat map represents value of the correlation coefficient ranging from 1 to −1, respectively. *p < 0.01, **p < 0.001, ***p < 0.0001.

For each 10-s tapping trial, an associated dwell and in-between tap time mapped out across two axes (Figure 4A). We removed *intra-individual* outlier points using the 1.5^*^ IQR threshold to eliminate spuriously long taps or in-between breaks (Figure 4A; crossed points). We then measured *sustained* irregularities in tapping speed and dwell time using the coefficient of variation as Cerebellar Dysfunction Score (CDS, Figure 4B).

The dominant hand CDS significantly correlated with neurological clinical scale scores for NeurEx cerebellar functions (*R* = 0.62, *p* < 0.0001) and the Kurtzke FSS cerebellar function score (*R* = 0.55, *p* < 0.001). But most importantly, when we construed a new measure, that compares cerebellar dysfunction to motor weakness (i.e., normalized, hand-specific NeurEx *cerebellar dysfunction (CD) score* divided by normalized, hand-specific NeurEx *strength score*; with 1 added to each to avoid dividing by 0) termed as the “CD vs. Motoric Score,” we validated that CDS identifies patients with cerebellar dysfunction that is in excess to motor weakness (Figure 4B). Specifically, CDS of the dominant hand significantly (*p* < 0.001) correlates with the CD vs. Motoric Score dominant (*R* = 0.60), while not correlating with any measure of dominant hand strength or corticospinal dysfunction (Figure 4D). In addition, based on HV cut-off (HV mean + 2SD), the CDS can differentiate MS patients into groups with and without cerebellar dysfunction disproportionate to motor weakness with borderline significance (*U* = 2.43, *p* = 2.00e−02; Figure 4C).

In contrast, the non-dominant hand CDS had comparative (*R* = 0.51, *P* < 0.01) correlations with non-dominant cerebellar and strength subcomponents of the NeurEx (Figure 4D).

##### Fatigue

We hypothesized that if the tapping test is performed with maximal effort, it may be possible to measure the slowing of the performance with time as a measure of motor fatigue.

**Figure 5:**
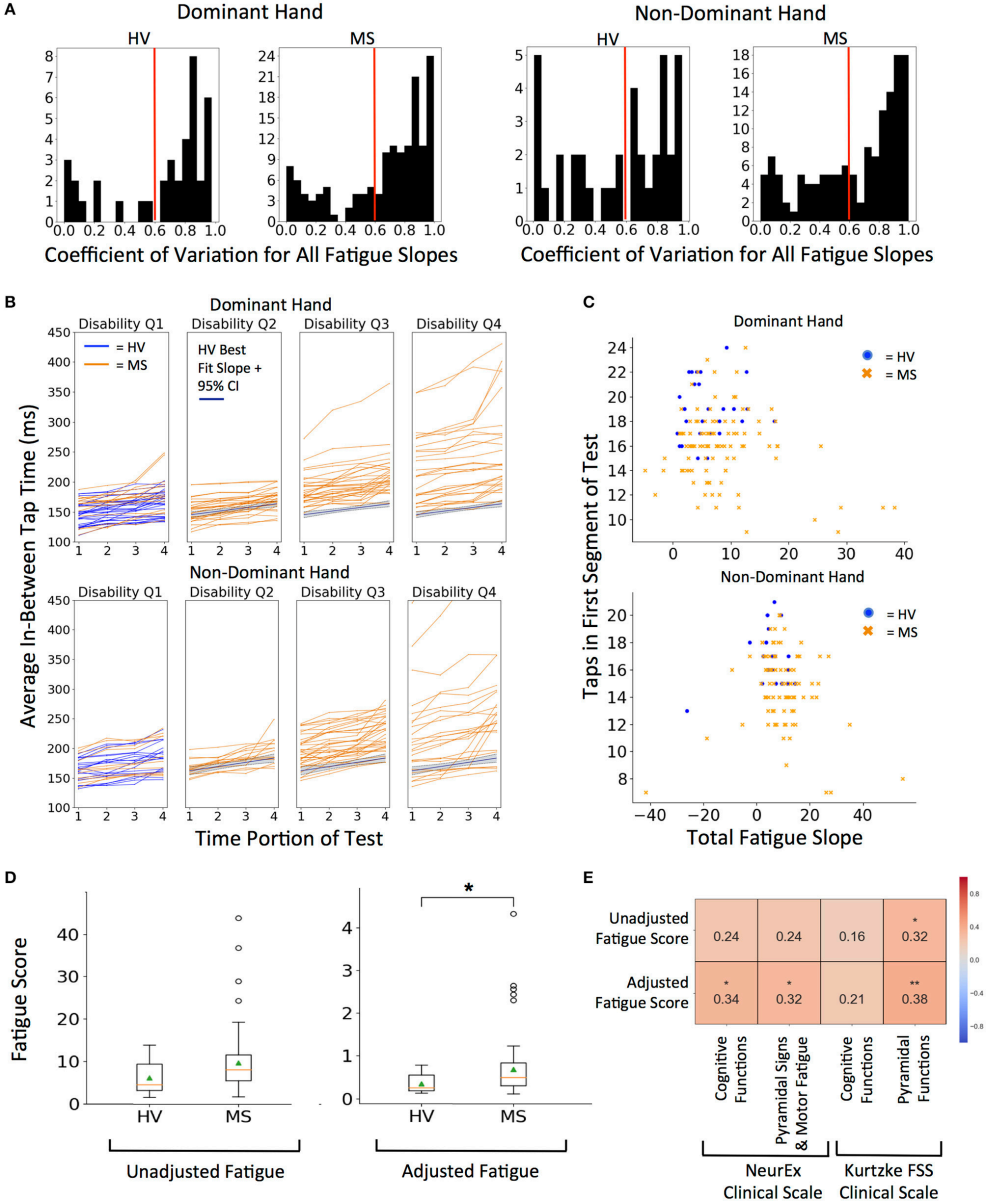
Quantifying fatigue through tapping. **(A)** Histograms for coefficient of variation for all fatigue slopes. Threshold of 0.6 R^2^ (marked with red line) was used as quality control for all future fatigue analysis. 0.8 R^2^ was used for sensitivity analysis in Supplementary Figures. **(B)** Distribution of fatigue slopes for HV (blue lines) and MS (orange lines) cohorts. MS patients are split into disability quartiles based on their total disability measured by NeurEx. The HV best fit fatigue slope with 95% confidence interval is overlaid in disability quartiles 2–4 for reference. **(C)** Fatigue Slope compared to starting taps to derive adjusted fatigue metric in HV (blue dots) and MS (orange dots) cohorts **(D)** Distribution of unadjusted and adjusted fatigue scores between cohorts. On each box plot, green triangles indicate the mean and orange lines indicate the median of depicted cohort datasets. Box plot box boundaries represent the Q1 to Q3 range centered about the median. Box plot whisker lengths extend to Q1–1.5 *IQR and Q3 + 1.5 *IQR. (**E)** Pearson correlations with corresponding clinical scores. Red to blue heat map represents value of the correlation coefficient ranging from 1 to −1, respectively. *p < 0.01, **p < 0.001.

To limit noise, we decided to average the quality-controlled (QC, i.e., eliminating *intra-individual* outlier taps) in-between tap times across 4 quartiles (each 2.5 s) of the 10-s intervals for each subject. We investigated elimination of the first and last seconds, but this step did not change the analysis and was abandoned. We then fitted intra-individual linear regression models across 4 quartiles to generate the “fatigue slope.” This fatigue slope may be clinically meaningful only if the model is reliable. Therefore, we implemented coefficient of determination (R^2^) cut-off for model reliability based on distribution of group data (Figure 5A). The generated histograms led to logical cut-off of 0.6 (i.e., the linear regression models explained at least 60% of variance in the patient-specific four quartiles). As a sensitivity analysis we also explored more stringent cut-off of 0.8, which led to analogous results (Supplementary Figure 2).

We explored two measures of fatigue: 1. Unadjusted fatigue slope (Figure 5B) and 2. Adjusted fatigue slope, where we tried to construe a measure that might, at least partially, adjust for the level of effort. We considered this necessary because our hypothesis assumed that subject performs the test with maximal effort. Submaximal effort may be sustained during the entire test duration, artificially lowering the fatigue slope. Indeed, we saw that rare subjects achieved negative fatigue slope. Because this is clinically impossible (by definition, motor fatigue implies decreased motor performance in time), we excluded these subjects from further analysis.

Thus, the adjusted fatigue slope was calculated as fatigue slope divided by the total number of taps in the first 2.5 s time portion (Figure 5C). As demonstrated in Figure 5D, adjusted fatigue slopes were significantly increased in the MS group (*U* = −2.86, *p* = 4.21e−03) while unadjusted slopes were increased with borderline significance in the MS group (*U* = −2.39, *p* = 1.70e−02). Distributions of MS patients' raw fatigue slopes across 4 disability quartiles (Figure 5B) shows that motor fatigue increases with the level of neurological disability. Specifically, we saw no significant differences in the fatigue slopes for MS patients in first disability quartile and progressively higher differences for MS patients with increasing disability.

Unfortunately, we have not collected data for any accepted fatigue scale. Therefore, the best we could do is to correlate our putative fatigue measures with NeurEx and Kurtzke cognitive and motor subscores, for which we found mild, but nevertheless significant correlations (Figure 5E). The adjusted fatigue score had a stronger and more significant correlation than un-adjusted fatigue slopes. Adjusted fatigue correlated significantly with the NeurEx and Kurtzke pyramidal scores (*R* = 0.32, *p* < 0.01 and *R* = 0.38, *p* < 0.001, respectively) and with NeurEx cognitive functions score (*R* = 0.34, *p* < 0.01).

##### Cognitive and visual dysfunction

While tapping speed is independent of visual input (i.e., even blind person can achieve high tapping scores), the performance of the balloon popping test depends both on the visual input and reaction time, in addition to motor skills that drive performance of the tapping test. Therefore, we hypothesized that we may be able to derive more specific measures of visual functions and reaction speed by comparing performance on balloon popping with the tapping speed.

**Figure 6:**
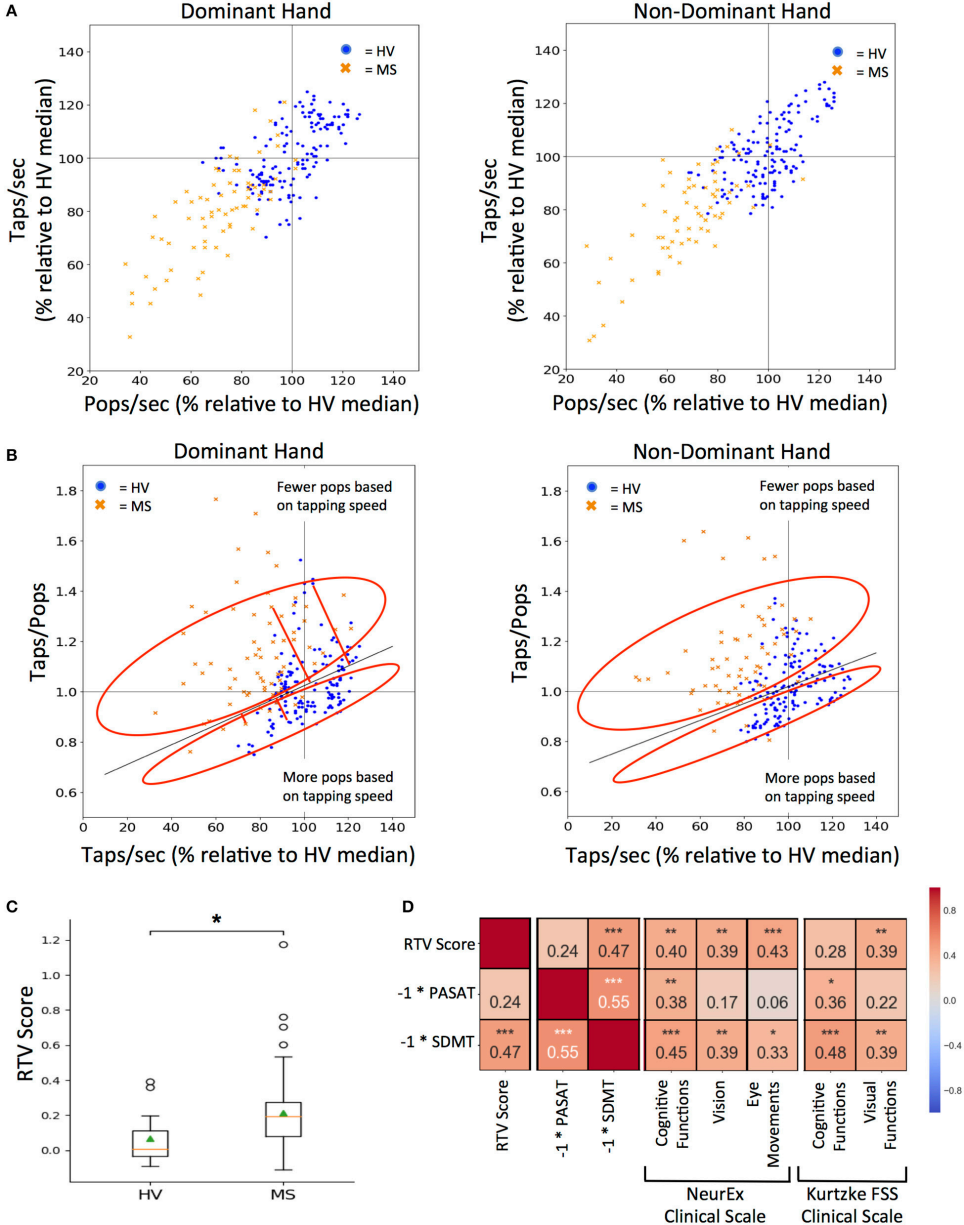
RTV: Response Time and Visual dysfunction score. **(A)** Tapping and popping scores directly compared for each test participant (blue dots for HV and orange dots for MS) on the first test sitting. 100% mark (gray lines) for both scores was derived from the median of all HV data for tapping and popping respectively. **(B)** Comparing each participant's taps/pops versus taps/sec to calculate RTV score, which is the orthogonal residual to the best-fit line (dark gray line) derived from HV data (cross sectional and longitudinal). The red circle vertically above the HV best fit line represents positive residuals which correspond to tests where the user had fewer pops based on their tapping speed as compared to HVs and vice versa for the red circle vertically below the HV best fit line. Red lines extending from the HV best-fit line indicate the orthogonal residual examples that are used as RTV scores. **(C)** Distribution of RTV scores between HV and MS cohorts after each participant's cross-sectional dominant and non-dominant scores were averaged. On each box plot, green triangles indicate the mean and orange lines indicate the median of depicted cohort datasets. Box plot box boundaries represent the Q1 to Q3 range centered about the median. Box plot whisker lengths extend to Q1–1.5 *IQR and Q3 + 1.5 *IQR. **(D)** Pearson correlations between RTV scores and corresponding clinical tests and clinical scale score for cognitive and visual function. Red to blue heat map represents value of the correlation coefficient ranging from 1 to −1, respectively. *p < 0.01, **p < 0.001, ***p < 0.0001.

To achieve this goal, we first had to standardize the outcomes from the two smartphone tests, because even though, during app design we have increased the time for balloon popping test in comparison to tapping test from 10 to 26 s to get similar total number of taps and pops, we still observed lower number of pops in comparison to taps, especially for MS subjects. Hence, we normalized both tests to taps per second (taps/sec) and pops per second (pops/sec). Additionally, raw scores for both tests were converted to a percentage of the HV median score for the respective test (Figure 6A).

A taps/pops score was then generated from normalized data for each test user. The higher taps/pops score indicates relative underperformance in the balloon popping test in comparison to tapping test and therefore may indicate visual dysfunction or decrease in reaction time. While this is true if the tapping speed is high, it may not be true when the tapping speed is low, either due to low effort or motoric dysfunction. Indeed, if the tapping speed is too low, then even slow reaction time in the balloon popping test may be sufficient to achieve comparable speed in both tests.

Therefore, we plotted taps/pops against the average taps/sec score for all HV data (i.e., all cross-sectional and longitudinal, Figure 6B) as we hypothesized (and validated) that the tap/pops metric will be positively correlated to the tapping speed (*R* = 0.35, *p* = 2.10e−05). In other words, the faster the person taps, the less reaction time (s) he has available in the balloon popping test to achieve comparable results. Consequently, the taps/pops metric must be adjusted for the tapping speed and the linear regression model we derived represents physiological lag of the balloon popping test in relationship to achieved tapping speed. We then calculated the orthogonal residual of each cross-sectional data point for individual-specific taps/pops versus taps/sec values (depicted by a single blue dot for HV and single orange cross for MS patients) against the HV linear regression line. The higher the residual (i.e., subject points located above the HV regression line) the greater the difference between the expected and observed lag in the balloon popping test after adjustment for motoric performance in the tapping test. Consequently, we call this residual the RTV score, as, according to our hypothesis, it integrates Reaction Time and Visual functions and should separate these from pure motoric dysfunction.

The RTV score is significantly higher in MS versus HV cohort (*U* = 3.21, *p* = 1.32e−03; Figure 6C). Although there is no clinical measure of reaction time available, we correlated RTV score with cognitive and visual subdomains of NeurEx and Kurtzke FSS, as well as with currently utilized cognitive tests in MS clinical trials, SDMT, and PASAT. The RTV score has a significant correlation of 0.47 with the inverse score for the SDMT (*p* < 0.0001), but not with the inverse PASAT scores. For the NeurEx clinical scale, the RTV score correlates comparably with cognitive functions (*R* = 0.40, *p* < 0.001), vision (*R* = 0.39, *p* < 0.001), and eye movements (*R* = 0.43, p < 0.0001). For the Kurtzke FSS scale, the RTV score correlates similarly with visual functions (*R* = 0.39, *p* < 0.001; Figure 6D).

#### The smartphone-based outcomes are intra-individually stable in short time intervals

One of the desired features of measuring neurological functions via smartphone apps is development and validation of sensitive outcomes that can track progression of disability intra-individually, in clinical trials and routine clinical practice.

The first condition that such outcomes must fulfill is specificity, or low variance of the measurement in the absence of disease progression. The second condition is sensitivity, or the ability to detect even small (but true) changes in disease progression.

**Figure 7:**
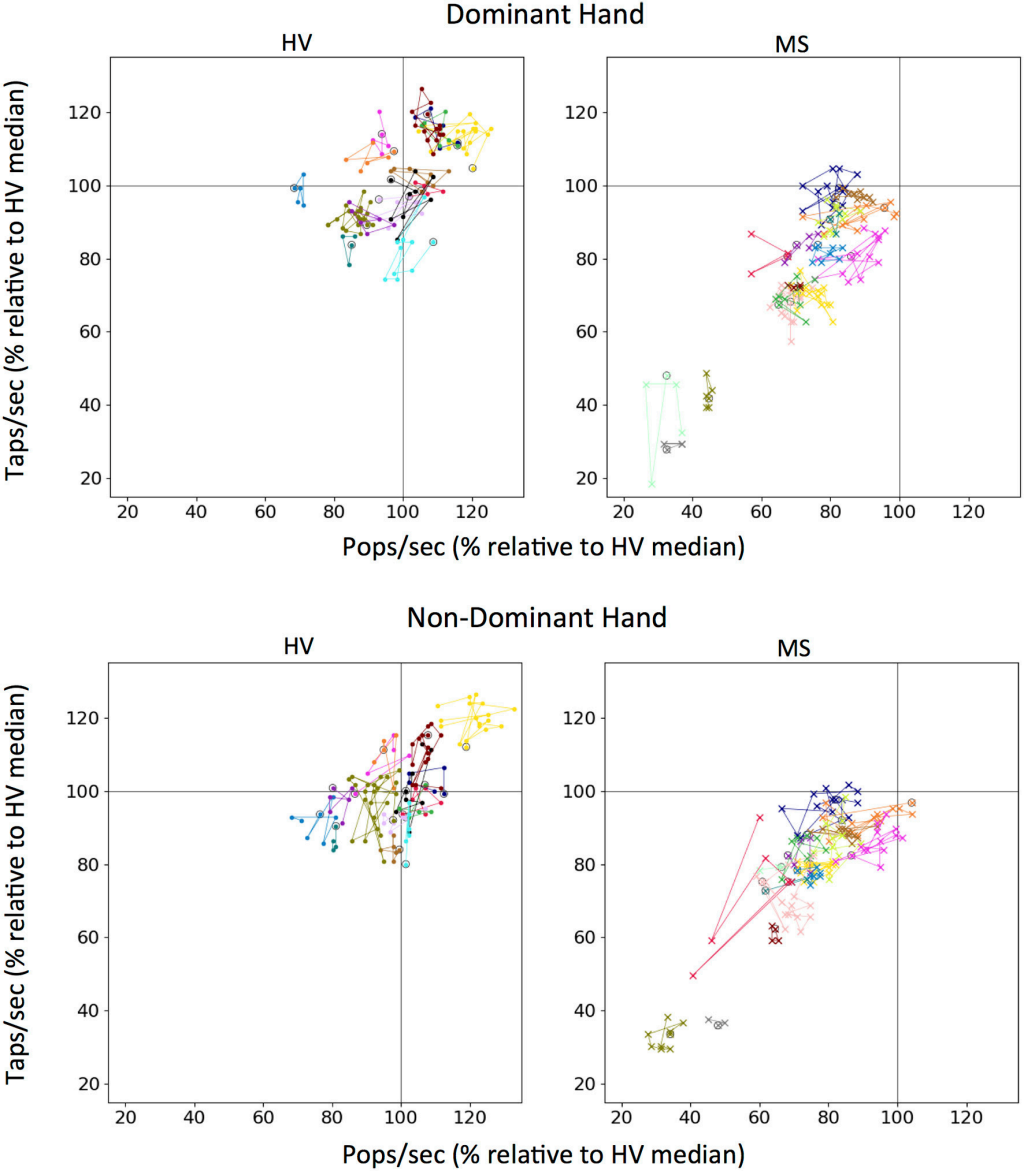
Longitudinal tapping test and balloon popping test data. Each color on a graph represents an individual testing participant and first test sitting is circled in black. Colors for unique individuals are consistent between dominant and non-dominant hand plots. One hundred percent mark (gray lines) for both scores was derived from the median of all Healthy Volunteer (HV) longitudinal data for tapping and popping respectively.

Because our longitudinal data comprise <3 months, we could evaluate only the specificity (or short-term longitudinal stability) question. The individual colors representing each patient are clustered (Figure 7). From the first test (indicated for each subject as circle), the change in tap score versus pop score can be monitored over time. None of the patients show a clear trend of improvement on either axis but distinct clusters can be visualized between patients. The MS cohort has clusters lower on both axes as compared to HV.

## Discussion

We observed generally stronger correlations between relevant clinician-derived scores with smartphone app scores than with 9HPT, and patients who could no longer perform 9HPT were still able to perform smartphone apps. Strong correlations between cumulative number of taps and pops with clinician-derived scores of cerebellar and motoric functions of upper extremities indicate that both smartphone app tests are measuring, analogously to 9HPT, predominantly fine motoric skills, which are strongly affected by cerebellar dysfunction. Proprioception also contributes to the examined smartphone tests, which is (based on correlation analysis) not true for 9HPT. Considering the benefit of higher “ceiling effect threshold,” we conclude that 9HPT can be replaced in EDSS plus or CombiWISE clinical scales by smartphone-derived finger tapping. There are several finger-tapping apps now freely available for both android and iOS platforms, that can be used by clinicians, patients or clinical trial investigators. However, to our knowledge, these apps have not been formally tested in MS cohorts.

Balloon popping test, though comparatively as simple and convenient as the tapping test, broadened neurological functions involved in test execution to eye-hand coordination and cognitive functions (specifically, reaction time). This broadening resulted in slightly increased power of detecting differences between HV and MS patients in comparison to tapping test. Furthermore, the relative intra-personal stability of these measures in short-term (<3 months) longitudinal testing suggests that these smartphone tests may provide additional advantages over 9HPT measured in the clinic, by generating more granular data that can be analyzed as repeated measures or period averages to limit noise and enhance sensitivity in detecting progression of neurological functions in clinical trials.

But smartphone apps provide additional advantages over de-facto analog measurement of a single outcome by traditional functional test like 9HPT: by digitalizing the entire test, interesting secondary outcomes can be derived and their clinical utility explored and validated. We have derived three such “secondary” outcomes from the two apps we investigated here: The putative measure of motor fatigue, the measure of cerebellar dysfunction that is in excess to motoric weakness, and the composite measure of reaction time and eye/hand coordination.

These would be considered “expert-derived” outcomes, because they were based on hypotheses that stem from understanding of how different neurological functions may contribute to the performance of selected tests. For example if a subject provides maximal effort on the test, then we hypothesized that this effort may not be sustainable for the entire duration of the test and that the decline in the top performance may represent an objective measure of fatigability. We encountered several problems while investigating this hypothesis: e.g., when we divided duration of the test into segments and calculated segment averages from which we then derived “fatigue slope,” we realized that some of these slopes are unreliable, because linear regressions explained low proportion of variance of measured values. One cannot derive reliable estimates from unreliable models, so we first had to introduce a quality measure: based on the distribution of *R*^2^-values we selected *R*^2^ > 0.6 as logical cut-off for data reliability. Within this cut-off we observed that our hypothesis was correct even for healthy volunteers: the mean time between the taps increased between individual segments, leading to positive slope that reflects decrease in performance. However, while examining healthy volunteer data we noticed another problem: how do we know that a subject performs the test with 100% effort? We observed positive fatigue slope in top performers, while some HVs with slower tap scores could maintain their, potentially sub-maximal, performance without obvious fatigue (i.e., had negative fatigue slopes). Therefore, we explored adjusting raw fatigue score for the speed of test performance. Both fatigue scores were found to be higher in MS patients in comparison to HVs, but adjusted fatigue score was significantly better. In MS group, we also observed inverse correlation between fatigue and tapping speed, indicating that MS patients with greater motoric disability also experience greater motor fatigue. This conclusion agrees with functional MRI observations that patients with greater motoric disability require activation of larger areas of the brain to achieve comparable motoric performance to less disabled patients or HVs (11).

Next, we asked whether we can separate motoric disability from cerebellar dysfunction based on the hypothesis that cerebellar dysfunction may lead to greater variance of time between taps when adjusting for the tapping speed (i.e., will have higher coefficient of variation in either dwell time, in-between tap time or both). It is hard to separate cerebellar and motoric dysfunction, because on a group level, they are significantly correlated in MS and because they both contribute to fine finger movements. Nevertheless, CDS achieved strong correlation with the cerebellar domain of neurological examination, and was also the only measured outcome that correlated significantly with the newly construed cerebellar versus strength feature of NeurEx. Indeed, patients who had elevated CDS above the healthy volunteer threshold also had significantly elevated cerebellar versus strength feature of NeurEx, indicating that in these patients, cerebellar dysfunction is greater than associated weakness. While this was true for the dominant hand, we did not see the same behavior in the non-dominant hand. We can only hypothesize why this is the case: perhaps the cortex and cerebellum exert lesser motoric control over the non-dominant hand, resulting in large variance of the tapping speed caused already by motoric weakness. In contrast, the dominant hand is trained for precise movements even under conditions of motoric weakness by its daily use. Under these circumstances, the cerebellum may compensate for the weakness by achieving precise and coordinated movements that are simply slow. Additional lesions in the cerebellar tracts take this control away, resulting in increased variance of tapping speed.

Finally, we explored combination of both tapping and balloon popping outcomes to derive feature that may better reflect reaction time. Our hypothesis was that tapping speed reflects pure motoric (strength and cerebellar) functions while balloon popping includes additional neurological functions such as eye/hand coordination and reaction time. Thus, lag of performance on balloon popping in comparison to finger tapping (in relationship to HV performance) should reflect these additional neurologic functions. Indeed, we found strong correlation of this novel metric with SDMT cognitive test, as well as cognitive subdomains of Kurtzke disability steps and NeurEx cognitive functions. We also found that visual acuity had lower effect on this metric than eye movement subscores of the digitalized NeurEx, demonstrating contribution of eye/hand coordination to the test performance.

This pilot data strongly supports our overarching hypothesis that combining multiple functional (smartphone-based) tests, rationally-designed to capture diverse neurological functions, may one day allow reconstruction of the entire neurological examination. Of course, achieving such goal will require developing and testing a variety of app-based tests in large cohorts of patients studied for longer time periods and independently validating resulting metrics. These efforts are currently ongoing in multiple research groups, including within pharmaceutical industry. Large cohorts will also allow development of secondary features of clinical utility using unbiased statistical (or machine) learning. Because models derived from these hypotheses-agnostic learning strategies tend to overfit the data, independent validation cohorts are necessary for translating these research efforts into clinical practice.

In conclusion, this study provides concrete examples of multiple advantages of smartphone-derived, digitally-encoded functional tests in comparison to current investigator-administered functional tests used in drug development for neurological diseases.

## Ethics statement

This study was carried out in accordance with the recommendations of the 45 CFR 46, NIH Combined Neuroscience Institutional Review Board. The protocols were approved by the NIH Combined Neuroscience Institutional Review Board. All subject gave written or digital informed consent in accordance with the Declaration of Helsinki.

## Author contributions

AB executed tests with patients in the clinic, completed data analysis and figures for app features, and contributed to initial app development and creation of app features. EK was responsible for the initial prototypes of app development and maintenance of the cloud-based databases. TH was responsible for app updates and maintenance of the software and cloud-based databases. PK exported and calculated the clinical scores that were used for correlations with NeurEx clinical scale. AW conducted neurological examinations for clinical trials participants and provided clinical scores used in correlations. MS conducted neurological examinations for clinical trials participants and provided clinical scores used in correlations. AM collaborated on the initial development of the app and provided software development expertise. BB developed the initial concept of the smartphone test suite containing the Tapping and Balloon Popping apps in addition to guiding all data analysis and feature development.

### Conflict of interest statement

The authors declare that the research was conducted in the absence of any commercial or financial relationships that could be construed as a potential conflict of interest.
