# Supplementary material for: Identifying and Quantifying Neurological Disability via Smartphone
Source: Front Neurol. 2018 Sep 4;9:740. doi: 10.3389/fneur.2018.00740 (PMC6131483; doi:10.3389/fneur.2018.00740)
Supplement: Supplementary file 1 [file Data_Sheet_1.DOCX]

Supplementary Material

Identifying and quantifying neurological disability via smartphone

Alexandra Boukhvalova, Emily Kowalczyk, Thomas Harris, Peter Kosa, Alison Wichman, Mary Alice Sandford, Atif Memon, Bibiana Bielekova*

*** Correspondence:** Bibiana Bielekova: Bibi.Bielekova@nih.gov

**
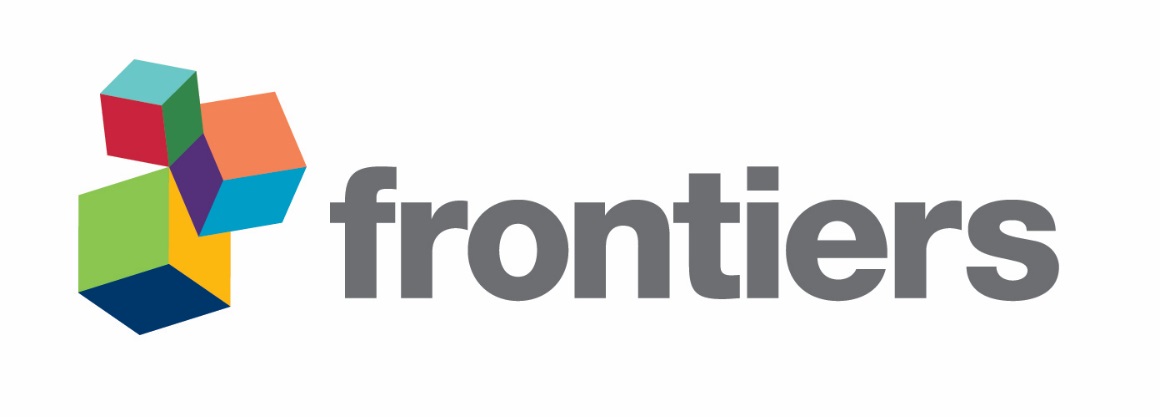
**

**Supplementary Figure 1**

**
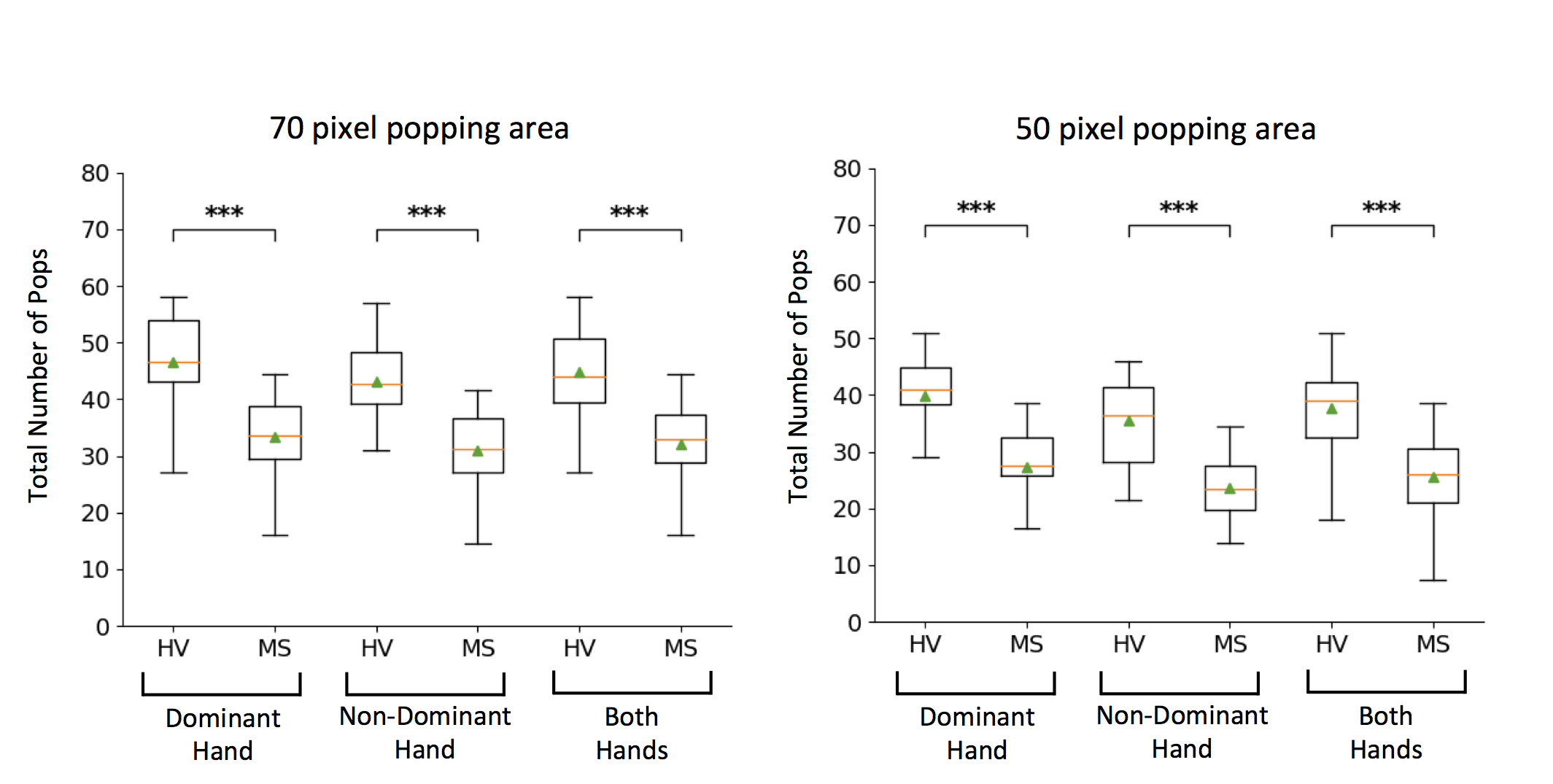
**

**Supplementary Figure 1.** Sensitivity analysis of Balloon popping tests with different sizes of the balloon: Healthy Volunteer (HV) and Multiple Sclerosis/Neurological Condition (MS) cohort comparison for raw scores on popping tests with variations of balloon size. Medium size popping area (left) and smallest size popping area (right) were tested against the largest, 100 pixel popping area used in the main total number of pops analysis. On each boxplot, green triangles indicate the mean and orange lines indicate the median of depicted cohort datasets. Boxplot box boundaries represent the Q1 to Q3 range centered about the median. Boxplot whisker lengths extend to Q1 – 1.5 * IQR and Q3 + 1.5 * IQR. ***p < 0.0001

**Supplementary Figure 2**

**
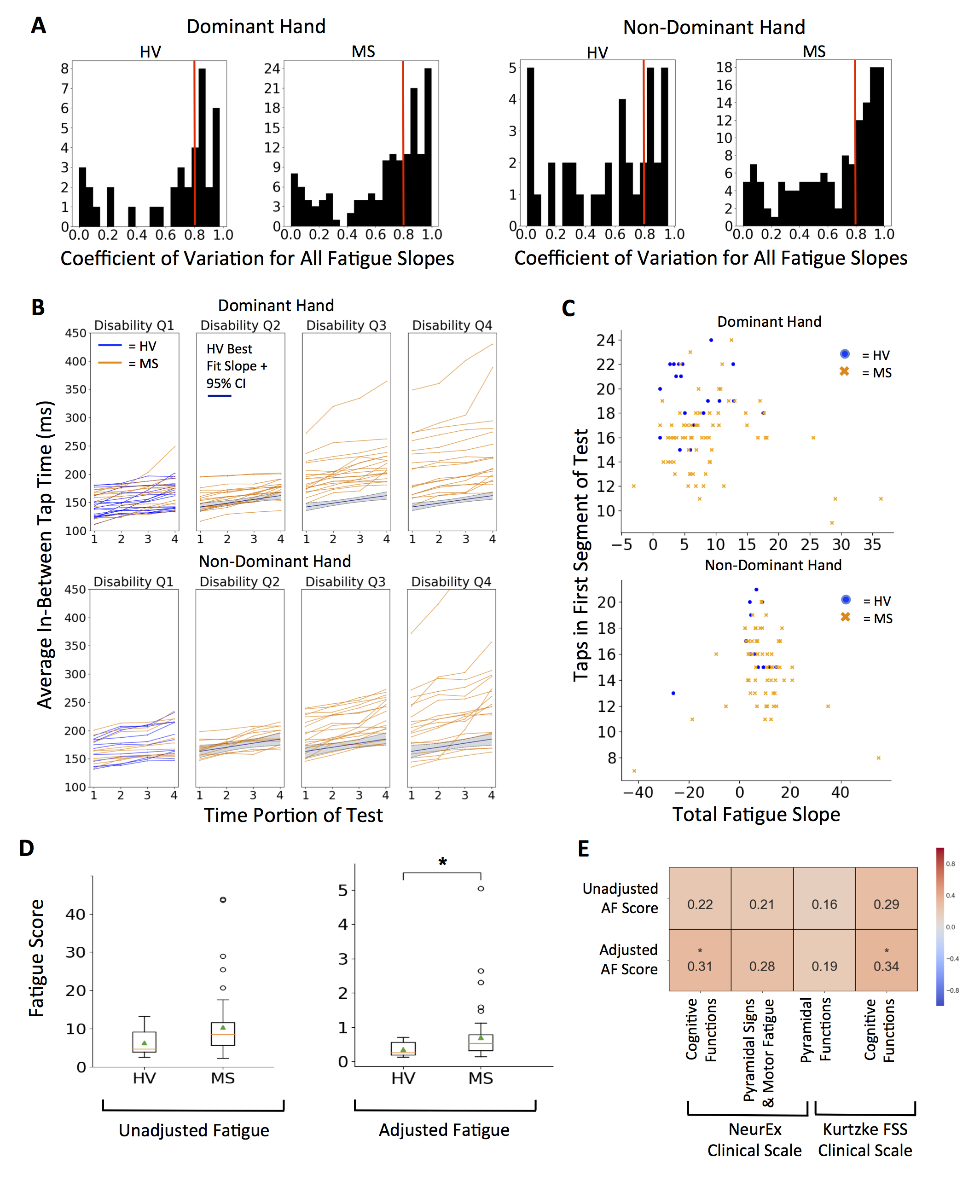
**

**Supplementary Figure 2.** Sensitivity analysis of R^2^ >0.8 threshold for reliability of fatigue slopes. **A.** Histograms for coefficient of variation for all fatigue slopes. Threshold of 0.8 R^2^ was used as further sensitivity analysis for fatigue score. **B.** Distribution of fatigue slopes for HV and MS cohorts. MS patients are split into disability quartiles based on their total NeurEx score. The HV best fit fatigue slope with 95% confidence interval is overlaid in disability quartiles 2-4 for reference. **C.** Fatigue Slope compared to starting taps to derive adjusted fatigue metric. **D.** Distribution of unadjusted and adjusted fatigue scores between cohorts. On each boxplot, green triangles indicate the mean and orange lines indicate the median of depicted cohort datasets. Boxplot box boundaries represent the Q1 to Q3 range centered about the median. Boxplot whisker lengths extend to Q1 – 1.5 * IQR and Q3 + 1.5 * IQR. **E.** Pearson correlations with corresponding clinical scores. Red to blue heat map represents value of the correlation coefficient ranging from 1 to -1, respectively. *p < 0.01

**Supplementary Video 1**

URL <https://youtu.be/5wFkbrvkodc>

**Supplementary Video 1.** Instructional video for finger tapping test. Patient is instructed to tap as quickly as possible over the grey region on the bottom portion of the screen for the 10-second duration of the test. Timer begins as soon as the patient starts tapping.

**Supplementary Video 2**

URL <https://youtu.be/a3ro1gYMYCw>

**Supplementary Video 2.** Instructional video for balloon popping test. Patient is instructed to press the Start button and tap on the randomly generated balloons until the 26-second trial is completed and balloons disappear.
